# Biogeography and molecular diversity of coral symbionts in the genus *Symbiodinium* around the Arabian Peninsula

**DOI:** 10.1111/jbi.12913

**Published:** 2017-01-02

**Authors:** Maren Ziegler, Chatchanit Arif, John A. Burt, Sergey Dobretsov, Cornelia Roder, Todd C. LaJeunesse, Christian R. Voolstra

**Affiliations:** ^1^ Division of Biological and Environmental Science and Engineering (BESE)Red Sea Research CenterKing Abdullah University of Science and Technology (KAUST)ThuwalSaudi Arabia; ^2^ Center for Genomics and Systems BiologyNew York University Abu DhabiAbu DhabiUnited Arab Emirates; ^3^ Department of Marine Science and FisheriesCollege of Agricultural and Marine SciencesSultan Qaboos UniversityMuscatOman; ^4^ Shelf Sea System EcologyAlfred Wegener Institute Helmholtz Centre for Polar and Marine ResearchGermany; ^5^ Department of BiologyPennsylvania State UniversityUniversity ParkPA16802USA

**Keywords:** coral reef, ecosystem, ITS2, next‐generation sequencing, Persian/Arabian Gulf, Red Sea, Sea of Oman, symbiosis

## Abstract

**Aim:**

Coral reefs rely on the symbiosis between scleractinian corals and intracellular, photosynthetic dinoflagellates of the genus *Symbiodinium* making the assessment of symbiont diversity critical to our understanding of ecological resilience of these ecosystems. This study characterizes *Symbiodinium* diversity around the Arabian Peninsula, which contains some of the most thermally diverse and understudied reefs on Earth.

**Location:**

Shallow water coral reefs throughout the Red Sea (RS), Sea of Oman (SO), and Persian/Arabian Gulf (PAG).

**Methods:**

Next‐generation sequencing of the ITS2 marker gene was used to assess *Symbiodinium* community composition and diversity comprising 892 samples from 46 hard and soft coral genera.

**Results:**

Corals were associated with a large diversity of *Symbiodinium*, which usually consisted of one or two prevalent symbiont types and many types at low abundance. *Symbiodinium* communities were strongly structured according to geographical region and to a lesser extent by coral host identity. Overall symbiont communities were composed primarily of species from clade A and C in the RS, clade A, C, and D in the SO, and clade C and D in the PAG, representing a gradual shift from C‐ to D‐dominated coral hosts. The analysis of symbiont diversity in an Operational Taxonomic Unit (OTU)‐based framework allowed the identification of differences in symbiont taxon richness over geographical regions and host genera.

**Main conclusions:**

Our study represents a comprehensive overview over biogeography and molecular diversity of *Symbiodinium* in the Arabian Seas, where coral reefs thrive in one of the most extreme environmental settings on the planet. As such our data will serve as a baseline for further exploration into the effects of environmental change on host–symbiont pairings and the identification and ecological significance of *Symbiodinium* types from regions already experiencing ‘Future Ocean’ conditions.

## Introduction

Reef‐building corals are the foundation of reef ecosystems and provide habitats to a diverse set of marine species, many of which are economically and ecologically important (Roberts *et al*., 2002). The ability of scleractinian corals to build reef structures critically relies on their ability to form symbioses with photosynthetic dinoflagellates of the genus *Symbiodinium* Freudenthal, 1962 (Muscatine & Porter, 1977). These intracellular algae provide up to 95% of the energy needs of the coral host (Falkowski *et al*., 1984). *Symbiodinium* species are ecologically diverse, exhibiting discrete associations with different coral hosts that can differ over large geographical scales, depth, season, and exposure to stressors (LaJeunesse *et al*., 2004, 2010; Finney *et al*., 2010; Ziegler *et al*., 2015). Furthermore, *Symbiodinium* species vary in their nutritional benefits to the hosts (Stat *et al*., 2008a; Cantin *et al*., 2009; Baker *et al*., 2013) and in their response to thermal stress and varying light intensity (LaJeunesse, 2001; Iglesias‐Prieto *et al*., 2004; Ziegler *et al*., 2014; Pettay *et al*., 2015). Hence, detailed knowledge of *Symbiodinium* coral pairings is arguably critical to our understanding of ecological resilience of coral reefs.

Of the currently nine clades of *Symbiodinium* (Pochon & Gates, 2010), the clades A, B, C, and D are most commonly associated with corals (Pochon *et al*., 2014). These clades can be further subdivided into subclades and types likely comprising hundreds of species. However, delineation of *Symbiodinium* diversity is not straightforward. Due to the deep phylogenetic divergence in the genus *Symbiodinium*, differences between clades can match differences observed at the level of order in other dinoflagellates (Rowan & Powers, 1992). Hence, no single universal marker gene exists to tease apart all ecologically discrete units (LaJeunesse, 2001; Pochon *et al*., 2014). Recent studies have applied specific multigene phylogenies to a single clade under study that successfully characterized distinct species and lineages (LaJeunesse & Thornhill, 2011; Lajeunesse *et al*., 2012). Despite the limitations of single gene phylogenies, the Internal Transcribed Spacer 2 (ITS2) region remains the most commonly used marker for *Symbiodinium* diversity typing. Because of the tandem repeat arrangement of rRNA genes, the ITS2 gene is a multicopy marker, which makes discerning inter‐ from intragenomic variation critical (Thornhill *et al*., 2007; Sampayo *et al*., 2009). Denaturing gradient gel electrophoresis (DGGE) can be used to address this issue by identifying the numerically dominant ITS2 variant(s) that in many cases represents a reproducible ITS2 ‘type’ that can be associated with the underlying dominant *Symbiodinium* species (LaJeunesse, 2001; Sampayo *et al*., 2009; Arif *et al*., 2014). However, DGGE lacks sensitivity to identify background symbiont types, especially if their abundance is < 5–10% (Thornhill *et al*., 2006; LaJeunesse *et al*., 2008). Bacterial cloning, in comparison, tends to overestimate ITS2 diversity by potentially amplifying genomically rare variants that may further complicate discrimination of intra‐ and intergenomic variation of ITS2‐based *Symbiodinium* diversity (Thornhill *et al*., 2007).

Recently, next‐generation sequencing (NGS) has been utilized for typing *Symbiodinium* ITS2 diversity, enabling the identification of distinct ITS2 types below 1% in abundance (Arif *et al*., 2014; Quigley *et al*., 2014; Thomas *et al*., 2014). High‐throughput sequencing of the ITS2 gene locus creates an opportunity to derive Operational Taxonomic Unit (OTU) cut‐offs via the assessment of average intragenomic diversities retrieved from the sequencing of isoclonal cultures that represent different species, as demonstrated by Arif *et al*. (2014). In the study by Arif *et al*. (2014), a range of isoclonal cultures representing different clades was sequenced and intragenomic ITS2 variance was successfully collapsed into distinct OTUs at a 97% similarity cut‐off. This approach was subsequently applied to field‐collected specimens where Arif *et al*. (2014) showed that this approach efficiently reduced the complexity of ITS2 NGS data and allowed for economic and efficient comparative analysis of a large number of coral species in a common and reproducible framework. Yet, sequence‐based analysis of NGS data faces some of the same challenges as bacterial cloning in discerning intra‐ from intergenomic ITS2 diversity. As a consequence, OTU‐derived *Symbiodinium* species diversity estimates must be considered provisional and can be overconservative for some specimens (but see Arif *et al*. (2014)). Thus, concomitant analyses of ITS2 sequence data in combination with an OTU‐based approach provide a means to interrogate NGS data in a manner that allows elucidating symbiont diversity of known *Symbiodinium* types and an assessment of taxon richness that addresses the challenges associated with a multi‐copy marker such as ITS2.

The seas surrounding the Arabian Peninsula, including the Red Sea (RS), the Sea of Oman (SO), and the Persian/Arabian Gulf (PAG), represent an understudied marine region despite hosting a large diversity of coral reef ecosystems (Riegl *et al*., 2012; Bauman *et al*., 2013; Coles *et al*., 2015). The RS is an oligotrophic system with high temperature variation and high salinity due to low influx of freshwater, high evaporation and limited exchange with the Indian Ocean (Sheppard *et al*., 1992). The environmental conditions in the PAG are arguably the most extreme in the world under which corals exist. Corals in the PAG are exposed to extreme fluctuations of temperatures (from 11 to 36 **°**C) and high salinity (often > 44 PSU) (Coles & Riegl, 2013). The conditions experienced by corals in the RS and the PAG are generally beyond the limits of what corals experience and survive elsewhere, which for the PAG has been shown to be partially attributable to a recently identified symbiont species, *Symbiodinium thermophilum* (Hume *et al*., 2015), that is prevalent in the PAG due to its preference to high salinity (D'Angelo *et al*., 2015). On the contrary, the temperature (22–32 **°**C) and salinity (> 37 PSU) conditions in the SO are less extreme than in the PAG, due to greater mixing with the wider Indian Ocean (Piontkovski & Al‐Jufaili, 2013).

To date, *Symbiodinium* diversity has been primarily studied in coral species from various locations in the Caribbean, the Central Pacific, and the Great Barrier Reef (LaJeunesse *et al*., 2004; Finney *et al*., 2010; Tonk *et al*., 2013), but data from the Arabian region are limited. Studying symbiont diversity in reefs of the RS and PAG might provide critical insight to our understanding of coral resilience and the underlying adaptations that allow corals to survive under future climate change scenarios. In this context, the more moderate conditions in the SO, comparable to other major coral reef habitats around the world, such as the Great Barrier Reef (Tonk *et al*., 2013), can serve an important baseline to disentangle geographically‐ from environmentally prompted patterns of *Symbiodinium* diversity and abundance. To provide a comprehensive assessment of coral‐associated *Symbiodinium* diversity in the seas around the Arabian Peninsula, we conducted next‐generation sequencing‐based ITS2 typing of 892 coral colonies representing 46 coral genera from the RS, SO, and PAG. Furthermore, we investigated *Symbiodinium* diversity and community structure within and between coral colonies and genera across regions.

## Materials and methods

### Sample collection and environmental conditions

**Figure 1:**
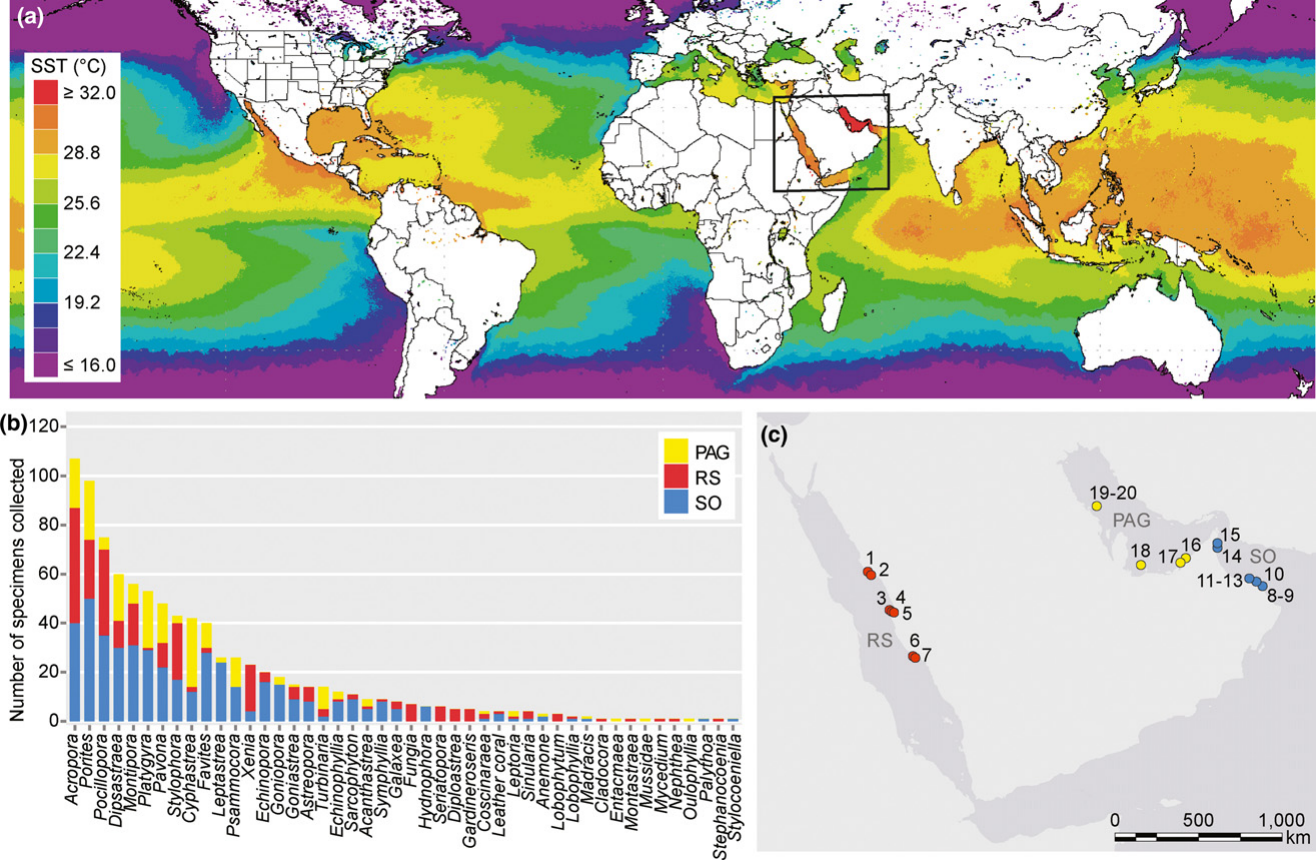
Overview of study locations and coral sampling. (a) Summer sea surface temperatures (SST) mark the region around the Arabian Peninsula as comparably warm (monthly averages from 07/2012–09/2012 derived from satellite data sets of the NASA Giovanni online data system, Ocean Colour Radiometry, MODIS‐Aqua 4 km, black rectangle denotes sampling region). (b) Cumulative number of coral samples per genus collected from the Red Sea (RS, red), the Sea of Oman (SO, blue), and the Persian/Arabian Gulf (PAG, yellow), total *n* = 892. (c) Sampling sites around the Arabian Peninsula; samples from the Red Sea were collected at Yanbu Ayona (1, 36 samples), Yanbu 23 (2, 31 samples), Shib Nazar (3, 43 samples), Al Fahal (4, 42 samples), Inner Fasr (5, 31 samples), Abu Lath Shallow Reef (6, 40 samples), and Al Lith South Reef (7, 33 samples). Collection sites in the Sea of Oman included Bandar Al Khayran (8, 90 samples), Shiekh Al Sifah (9, 87 samples), Fahal Island (10, 62 samples), Daymaniyat Islands (11–13, 35, 36, and 58 samples), Al Aqah (14, 34 samples), Dibba Rock (15, 37 samples), and in the Persian/Arabian Gulf Ras Ghanada (16, 26 samples), Saadiyat (17, 42 samples), Al Yassat (18, 33 samples), Subri (19, 73 samples), and Chandelier (20, 23 samples). [Colour figure can be viewed at wileyonlinelibrary.com]

A total of 892 specimens of corals representing 46 genera were collected in the RS (January, February, and March 2014), the SO (August 2011, May 2012, and September 2012), and the PAG (May 2011 and September 2012) by SCUBA at depths between 3–15 m (Fig. 1, see Appendix S1 for sample overview). Due to logistical reasons, it was not possible to collect samples from different regions at the same time; however, most *Symbiodinium* communities are considered stable (Thornhill et al., 2006; Hume *et al*., 2015). All specimens were photographed for identification before sampling. Sampled specimens were stored in Ziploc plastic bags upon collection and transported in coolers filled with seawater. Upon returning to shore, samples were transferred into cryotubes, DMSO/NaCl buffer was added (Gaither *et al*., 2011), and samples were stored at 4 **°**C until DNA extraction.

### DNA extraction and PCR

DNA from all samples was extracted with Qiagen DNeasy Plant Mini Kit (Qiagen, Hilden, Germany) following the manufacturer's protocol with minor modifications. Briefly, 100 mg of sample was added to 1.5‐mL Eppendorf tubes containing 500 μL sterile glass beads (BioSpec, Bartlesville, OK) and lysis buffer. Samples were homogenized with Qiagen tissue Lyzer II (Qiagen, Hilden, Germany) for 1 min and DNA extractions were continued according to manufacturer's instructions. All DNA samples were quantified with Qubit broad range DNA assay (Invitrogen, Carlsbad, USA) and normalized to 30 ng μL^−1^ for subsequent PCR reactions.

The PCR amplification of the ITS2 gene marker was performed using primers ITSintfor2 (LaJeunesse, & Trench, 2000) and ITS2‐reverse (Coleman *et al*., 1994). For the 454 platform, the primers included 454 LibL library adapters and an 8‐bp barcode; for the MiSeq platform, primers included overhang adapters (Hume *et al*., 2016). PCRs were run in triplicate per sample with 12.5 μL of Qiagen Multiplex PCR Kit (Qiagen, Hilden, Germany), 0.1 μM primers, 30 ng DNA and a final volume adjusted to 25 μL with DNase‐free water. The following PCR conditions were used: 15 min at 94 °C, followed by 35 (454) or 27 (MiSeq) cycles of 94 °C for 30 s, 51 °C for 30 s, 72 °C for 30 s and a final extension step of 10 min at 72 °C. PCR products were run on a 1% agarose gel stained with 1× SYBR Safe (Invitrogen, Carlsbad, CA) to visualize successful amplification. For each sample, the triplicate PCR products were pooled.

### 454 ITS2 sequencing

DNA concentrations were measured with Qubit broad range DNA assay. About 8 ng of each pooled sample (see above) were combined for sequencing and ran on a 1% agarose gel to remove excess primers. The gel band was excised, purified with the Qiagen MinElute Gel Extraction Kit (Qiagen, Hilden, Germany), quantified with Qubit, and quality checked via Bioanalyzer (Agilent, Santa Clara, CA). About 100 ng of each pooled library was submitted to Macrogen (Korea) for sequencing using Titanium FLX chemistry. Samples from the RS were sequenced on two 454 half picotiter plates and produced 934,080 (mean length = 313.31 bp) and 948,659 (mean length = 309.66 bp) reads, respectively. In addition, samples from the SO were sequenced on two quarter 454 picotiter plates and produced 322,527 (mean length = 314.30 bp) and 310,441 (mean length = 310.05 bp) reads, respectively, and one library consisting of samples from the southern PAG was sequenced on a quarter 454 picotiter plate and yielded 325,252 reads (mean length = 318.20 bp).

### MiSeq ITS2 sequencing

Because of advances in NGS technology, some samples for this study were sequenced on the MiSeq platform (see Appendix S1 in Supporting Information). Pooled samples were cleaned with Agencourt AMPure XP magnetic bead system (Beckman Coulter, Brea, CA, USA). Nextera XT indexing and sequencing adapters were added via PCR (8 cycles, total PCR cycles for all samples = 35) following the manufacturer's instructions. The samples were then quantified on the Bioanalyzer and Qubit and pooled in equimolar ratios. The pooled library was purified on a 2% agarose gel to remove excess primer and sequenced at 8pM with 10% phiX on the Illumina MiSeq, 2 × 300 bp paired‐end version 3 chemistry according to the manufacturer's specifications. Samples from 96 corals from the northern PAG (samples AGa1‐AGc162, Appendix S1) were sequenced on the MiSeq Illumina platform and produced 6,472,783 paired‐end reads with an average read length of 331.69 bp.

### Next‐generation sequencing data processing

The sequences from each 454 library were processed according to the pipeline detailed in Arif *et al*. (2014). Briefly, sequencing reads were de‐noised using pyronoise (Quince *et al*., 2011), forward primer and barcode sequences were removed by the *trim.seqs* function in mothur 1.34.4 (Schloss *et al*., 2009), and low‐quality sequences were discarded according to the following criteria: barcodes (> 0 mismatches), forward primer (> 2 mismatches), ambiguities (> 0 bp), homopolymers (> 6 bp), and short sequence length (< 250 bp). Reverse primer sequences were removed with cutadapt 1.1 (Martin, 2011) and the overall error rate was set to 0.15. All identical sequences were subsequently collapsed and representative sequences were retained via *unique.seqs* command in mothur. Chimera sequences were removed with uchime as implemented in mothur (Edgar *et al*., 2011).

Paired‐end sequences from the Illumina MiSeq platform were merged using the *make.contigs* command in mothur. Forward and reverse primers were trimmed with cutadapt (Martin, 2011). Sequencing reads were quality trimmed with *screen.seqs* command and checked for chimeras with *chimera.uchime* in mothur.

**Figure 2:**
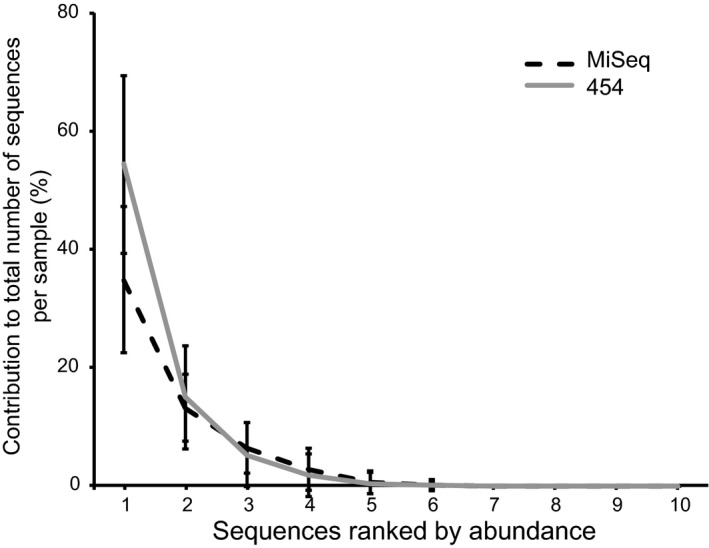
ITS2 sequence diversity contribution of the 10 most abundant ITS2 variants to total sequence composition in each coral sample from the seas around the Arabian Peninsula. Corals are dominated by the one or two most‐abundant ITS2 variant(s), error bars = SD.

Quality‐filtered reads from all libraries (i.e. 454 and MiSeq) were combined, redundant reads were collapsed (via *unique.seqs* function), and singletons were removed via *split.abund* function. The reads were then annotated to their respective ITS2 types against a custom ITS2 BLAST database (Arif *et al*., 2014) and 977 non‐*Symbiodinium* sequences were removed. The overall distribution of ITS2 sequences within coral colonies was assessed by considering the per cent contribution of the 10 most abundant ITS2 sequences per sample (irrespective of ITS2 identity) and averaging over the whole data set (Fig. 2).

### Sequence‐based ITS2 analysis

**Figure 3:**
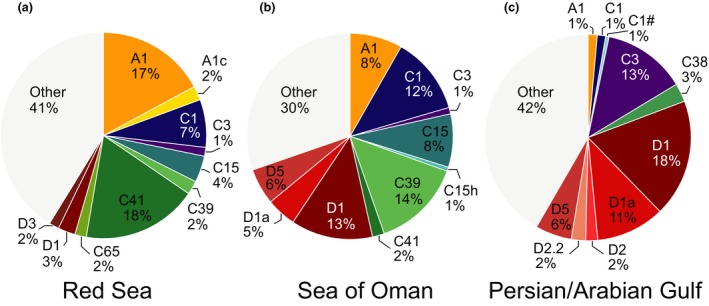
Signatures of ten most abundant *Symbiodinium* ITS2 types in each region. Overview is based on 892 samples comprising 46 hard and soft coral genera. (a) Red Sea (256 samples, 35 genera), (b) Sea of Oman (439 samples, 33 genera), (c) Persian/Arabian Gulf (197 samples, 24 genera). [Colour figure can be viewed at wileyonlinelibrary.com]

**Figure 4:**
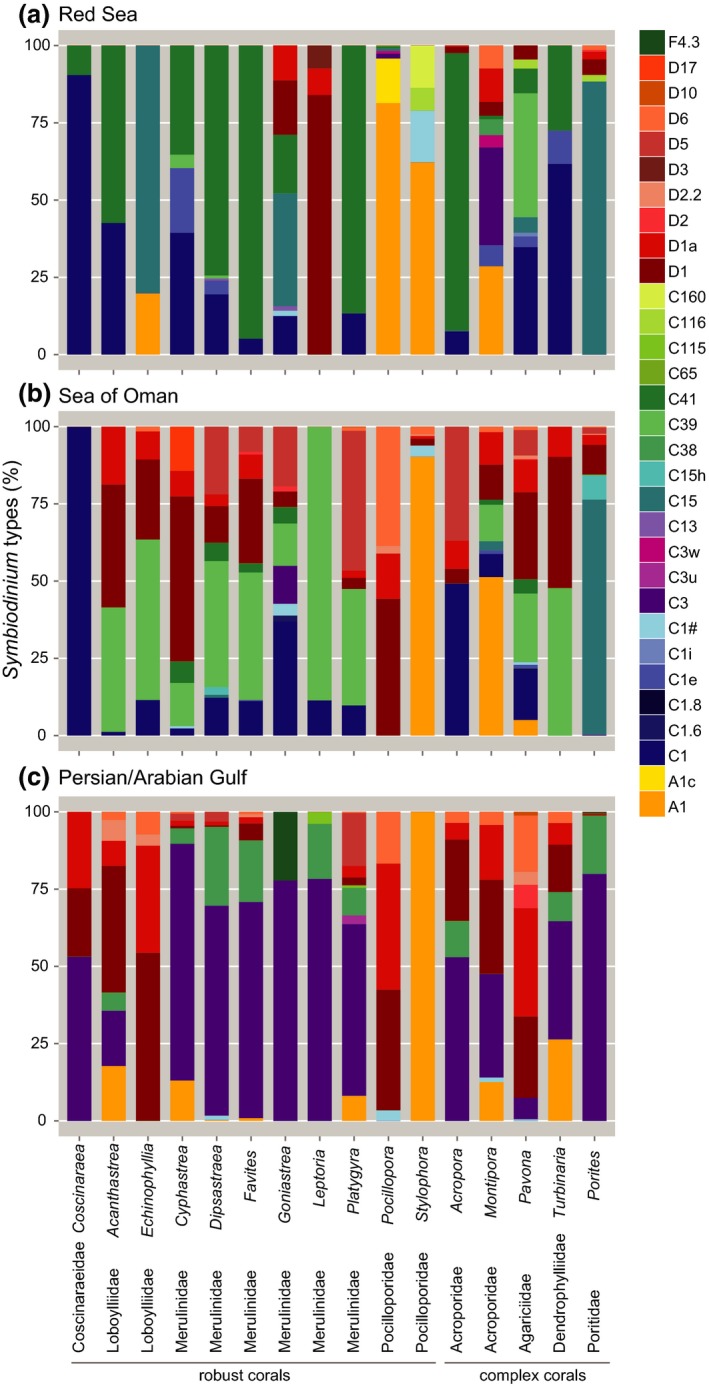
*Symbiodinium* ITS2 type‐based diversity associated with coral genera from the Arabian Peninsula. (a) the Red Sea, (b) the Sea of Oman, and (c) the Persian/Arabian Gulf. Only those sequences were included that represented ≥ 5% in abundance in at least one sample. Only coral host genera are displayed for which samples were available from all three regions. [Colour figure can be viewed at wileyonlinelibrary.com]

The 10 most abundant ITS2 sequences per region were compiled by counting over all samples for that region and differences in their respective contributions were visualized using pie charts (Fig. 3). To interrogate ITS2 data at different levels of resolution and facilitate comparison with previous studies using DGGE, we assessed the number of distinct ITS2 sequences that were present at a minimum cut‐off of ≥ 1% and ≥ 5% in at least 1 of the 892 samples (Table 1). Stack column charts detailing ITS2 composition over regions and genera were generated by averaging ITS2 count data of samples from the same host genera and region (Fig. 4).

**Table 1:** Sampling sites, number of samples and number of coral genera collected from the Red Sea, the Sea of Oman and the Persian/Arabian Gulf. The number of reads from 454 and MiSeq combined are indicated as well as the total number of ITS2 variants, numbers of ITS2 variants present at a minimum abundance of ≥ 1% or ≥ 5% in at least one sample, and number of OTUs per region

Regions	*n*	no. of coral genera	no. of sequence reads	no. of ITS2 variants	no. of ITS2 variants at ≥ 1% abundance	no. of ITS2 variants at ≥ 5% abundance	no. of OTUs subsampled to (1,000 sequences/sample)
Red Sea (7 reefs)	256	35	1,539,047	24,818	477	155	63
Sea of Oman (8 reefs)	439	33	549,259	14,337	352	136	39
Persian/Arabian Gulf (5 reefs)	197	24	5,734,486	95,710	256	128	23
ALL	892	46	7,822,792	118,205	977	223	92

### OTU‐based ITS2 analysis

For the OTU‐based analysis, ITS2 sequences were subsampled to 1,000 reads for each sample using the *sub.sample* command in mothur. The sequences within each clade were then aligned with muscle (Edgar, 2004), trimmed to equal length, and only sequences with a length ≥ 90% of all reads were retained. Remaining sequences were clustered at 0.03 cut‐off following the pipeline detailed in Arif *et al*. (2014) and OTUs were designated as OTUA1, OTUA2, …, OTUB1, OTUB2, …, to OTUG1 in order of decreasing abundance within each clade. The most abundant sequence for each OTU was chosen as the representative sequence for that particular OTU. OTUs were assigned to their corresponding ITS2 types via BLASTN against a custom ITS2 BLAST database (Arif *et al*., 2014).

Shared OTUs between the three regions (RS, SO, PAG) were obtained using Venn diagrams in mothur (command: *venn*). Next the composition of OTUs over all samples in each region was compared based on their annotation to *Symbiodinium* types. Statistical analyses of the OTU community composition were conducted on the seven coral genera for which at least three replicates were available from each region (i.e. *Acropora*,* Dipsastraea*,* Montipora*,* Pavona*,* Pocillopora*,* Porites*, and *Stylophora*, total *n* = 393). Symbiont community composition was investigated using permutational MANOVA (PERMANOVA) based on Bray–Curtis distances with the primer‐e 6 (PERMANOVA+) software package (Clarke & Gorley, 2006). For this analysis, OTU abundance data was log(*x* + 1) transformed and differences between the factor ‘region’ (3 levels: RS, SO, PAG) and the factor ‘reef’ (nested within ‘region’) were tested using partial sum of squares and 9,999 permutations under a reduced model. The average similarity of the *Symbiodinium* community in each coral genus was calculated per region using similarity percentage (SIMPER) analysis. Based on the number of OTUs in each sample of the seven host genera, we tested differences in OTU richness between regions using Kruskal–Wallis H test on ranks over all genera and for each genus separately (statistica 10, StatSoft Inc. 2011).

## Results

### Study overview and ITS2 sequence diversity

We collected 892 samples comprising 841 stony coral and 51 soft coral specimens from the RS, SO, and PAG representing corals from 46 different genera to assess *Symbiodinium* diversity around the Arabian Peninsula (Fig. 1, Table 1, see Appendix S1). Corals from 35 genera were collected from the RS, corals from 33 genera from the SO, and corals from 24 genera from the PAG. The six most common hard coral genera collected across all three regions were *Acropora*,* Dipsastraea*,* Montipora*,* Platygyra, Pocillopora* and *Porites,* which together represented half (*n* = 449, 50.34%) of the total number of specimens collected.

A total of 7,822,792 high‐quality sequence reads representing 118,205 distinct ITS2 sequences were generated and analysed (Table 1, Appendices S2 and S3). Although we identified over 100,000 distinct *Symbiodinium* ITS2 sequences in our data, only 977 ITS2 sequences were present at ≥ 1% abundance in at least one sample and 223 ITS2 sequences were present at ≥ 5% abundance in at least one sample (see Appendix S4), demonstrating the disparity between the total number of ITS2 sequences recovered and their relative proportion. This disparity was further substantiated by the distribution of ITS2 variants within individual coral colonies. In our data set, specimens were typically associated with one or two dominant ITS2 sequences that contributed on average 67% of all sequences followed by varying contributions from low abundant ITS2 variants (Fig. 2, see Appendix S3). This highly uneven read distribution of distinct ITS2 sequences (i.e. few ITS2 sequences at high abundance, very many at low abundance) was previously shown by Arif *et al*. (2014) and Thomas *et al*. (2014) and likely constitutes intragenomic ITS2 variants (or potentially, but less likely, rare background symbionts).

Samples from the PAG that were partially sequenced with MiSeq technology retrieved many more distinct ITS2 variants (95,710) than samples sequenced with the 454 technology (RS 24,818; SO 14,337) (Table 1). The increased ITS2 diversity, however, subsides when considering ITS2 variants that are present with ≥ 1% or ≥ 5% abundance in any sample (Table 1). This is highlighted by the lower number of distinct ITS2 variants at ≥ 1% and ≥ 5% in the PAG in comparison to the RS demonstrating that the increased ITS2 diversity via MiSeq sequencing does not affect the detection of the most abundant ITS2 variants. For this reason, we chose to consider only ITS2 variants that were present with at least 5% abundance in at least one sample for ITS2 type‐based analyses to facilitate comparative analysis of ITS2 diversity with results from previous studies using DGGE analysis and to avoid taking intragenomic diversity into account.

### ITS2 sequence‐based analysis of coral‐associated *Symbiodinium* in the Arabian Seas

To provide an overview over ITS2 sequence community structure, we looked at the *Symbiodinium* community composition across sampling sites by taking the 10 most abundant ITS2 sequences of each region (i.e. RS, SO, PAG) into account (Fig. 3). This analysis illustrates that all regions are dominated by ITS2 sequences from clades A, C, and D. Furthermore, the 10 most abundant ITS2 sequences encompassed about two‐thirds of all sequences (RS 59%, SO 70%, PAG 58%). At the same time, ITS2 sequence community structure was pronouncedly distinct between sites and every regional sea represented a unique signature. The RS was dominated by ITS2 sequences from clades A (19%) and C (35%) with a small contribution from clade D (4%). By comparison, in the SO, ITS2 sequences were largely dominated by clade C (38%) and higher contribution of clade D (23%), while ITS2 sequences from clade A contributed only 8%. In the thermally extreme PAG, clade D dominated the relative proportion of the 10 most abundant ITS2 sequences (39%), followed by ITS2 sequences from clade C (18%), whereas ITS2 sequences from clade A were rare (1%). Taken together, going from the RS over the SO to the PAG, we see a symbiont community shift that is represented by a decreasing contribution of ITS2 sequences from clade A, a stable contribution of ITS2 sequences from clade C (although it decreased in the PAG), and an increasing contribution of symbionts belonging to clade D. Our data show that *Symbiodinium* of coral reef communities dominating each region are specific and distinct from each other.

Next, we wanted to understand *Symbiodinium* community composition within coral genera across the RS, SO, and PAG. To do this, we focused on the 16 coral genera in our data set that were sampled across all three regions, and we considered only those ITS2 sequences that were present at ≥ 5% in at least one sample (Fig. 4). For the majority of host genera, the composition of the dominant *Symbiodinium* ITS2 sequences differed between the three regions and largely corresponded to the geographical pattern outlined above. For instance, *Acanthastrea* and *Echinophyllia* (both family Lobophylliidae), *Cyphastrea*,* Dipsastraea*,* Goniastrea*,* Favites* and *Platygyra* (all Merulinidae), *Acropora* (Acroporidae), *Pavona* (Agariciidae), and *Turbinaria* (Dendrophylliidae) associated mainly with *Symbiodinium* from clade C in the RS, whereas in the SO these coral genera harbored a mix of clade C and D (Fig. 4, see Appendix S4). In the PAG, depending on coral genus, *Symbiodinium* from clade D and/or *Symbiodinium* C3 were prevalent and *Goniastrea* additionally harbored *Symbiodinium* from clade F (F4.3, Fig. 4).

Interestingly, some coral genera diverged from this general pattern of geographical host–symbiont association. *Leptoria* (Merulinidae) only contained *Symbiodinium* from clade D in the RS, whereas it was only associated with *Symbiodinium* from clade C in the SO and PAG, and *Montipora* (Acroporidae) contained nine *Symbiodinium* types in the RS. Of these types only *Symbiodinium* A1 was commonly found in other genera in this region. Two other genera, *Porites* (Poritidae) and *Coscinaraea* (Coscinaraeidae), showed consistent *Symbiodinium* profiles between the RS and the SO, which diverged from the PAG (Fig. 4). Besides these region‐specific patterns, some coral genera showed more consistent associations with *Symbiodinium* between the RS, SO, and PAG. Colonies of *Stylophora* (Pocilloporidae) were consistently dominated by *Symbiodinium* A1 in all three regions (Fig. 4). *Pocillopora* (Pocilloporidae) was also dominated by *Symbiodinium* A1 in the RS, while colonies in both the SO and PAG, contained mixtures of *Symbiodinium* clade D (Fig. 4).

Similar to hard corals, soft corals were associated with different dominant *Symbiodinium* types at different locations. In the RS and SO, *Symbiodinium* communities in soft corals were composed of clade C and D, and all soft corals collected from the PAG shared the same dominant *Symbiodinium* type (C3), and hence, followed the pattern of prevalence of this symbiont type in the PAG as detected for the hard corals (Fig. 4).

### OTU‐based analysis of *Symbiodinium* community structure across regions and hosts

**Figure 5:**
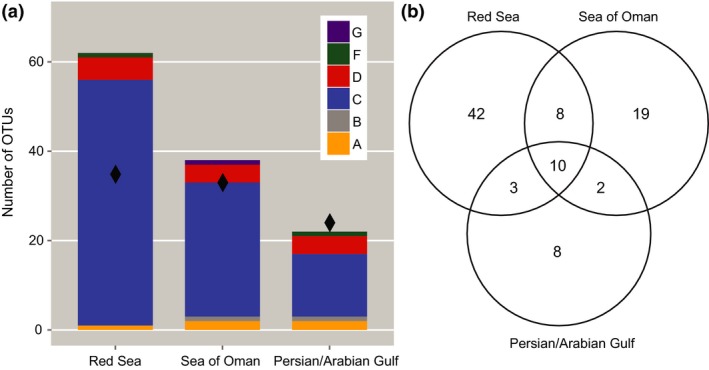
*Symbiodinium* OTU diversity in coral hosts around the Arabian Peninsula. (a) Stackplot illustrating OTU‐based clade diversity/composition over the total number of coral genera samples in each region (black diamonds) (b) Venn diagram showing number of *Symbiodinium* OTUs found in the Red Sea, the Sea of Oman, and the Persian/Arabian Gulf as well as number of OTUs that are shared between them. [Colour figure can be viewed at wileyonlinelibrary.com]

To further elucidate host–symbiont patterns and diversity across regions in a comparative framework, we derived OTUs on subsampled specimens. For OTU‐based analyses, data were subsampled to 1,000 sequences per sample resulting in the removal of 199 samples. In total, the subsampled data set comprised 693 samples across the RS, SO, and PAG representing 28,929 distinct ITS2 sequences. These distinct ITS2 sequences clustered into 92 OTUs (clade A = 3 OTUs, clade B = 1 OTU, clade C = 76 OTUs, clade D = 9 OTUs, clade F = 2 OTUs, and clade G = 1 OTU) at a 97% similarity cut‐off (Fig. 5a, see Appendix S5). The RS harboured the highest diversity of *Symbiodinium* OTUs (*n* = 63), most of which could be assigned to clade C (55 OTUs). In comparison, coral communities from the SO were comprised of 39 OTUs, most of which also could be assigned to clade C (30 OTUs). Only 23 *Symbiodinium* OTUs were determined for corals from the PAG, most of which belonged to clade C (14 OTUs). In comparison to the number of ITS2 types, our OTU approach indicated that the PAG may represent a stronger selected environment with comparably lower diversity than the RS and the SO (Table 1). Interestingly, richness of OTUs in clade C decreased between regions from 55 clade C OTUs in the RS to 30 and 14 OTUs in the SO and PAG, respectively, while richness in the other clades remained similar (Fig. 5a).

Among the 92 OTUs, 10 OTUs were shared between all regions (1 OTU in clade A, 6 OTUs in clade C, 3 OTUs in clade D) and an additional 8 OTUs were shared between the RS and the SO (7 OTUs in clade C, 1 OTU in clade D), while only 3 OTUs were shared between the PAG and RS (all 3 OTUs in clade C) and 2 OTUs between the PAG and SO (1 OTU each from clade B and C), respectively (Fig. 5b). This denotes that a large proportion of all OTUs was unique to each region (RS = 67%, SO = 49%, PAG = 35%). At the same time, the 10 shared OTUs together represented > 99% of all sequence reads.

We conducted an OTU‐based community analysis between regions taking data from the seven host genera into account that were present at least thrice in each region. These genera were *Acropora*,* Dipsastraea*,* Montipora*,* Pavona*,* Pocillopora*,* Porites*, and *Stylophora* comprising a total of 393 coral colonies (Table 2). The *Symbiodinium* community structure of these seven host genera aligned with geographical distance across the three regions. More specifically, we did not find a significant difference between OTU community structures of corals from the SO and PAG, but both were significantly different from the RS (PERMANOVA, factor ‘region’, *F* = 4.25, *P *<* *0.01, both pairwise comparisons *P *<* *0.05). OTU community structure for the factor ‘reef’ nested in ‘region’ was also highly significantly different (PERMANOVA, *F* = 5.95, *P *<* *0.001). Interestingly, pairwise tests between reefs within each region revealed only small differences between some of the reefs in the RS and SO, while the reefs in the northern PAG were clearly separated from reefs in the southern PAG (Fig. 1, see Appendix S6 for post‐hoc comparisons).

**Table 2:** Summary of *Symbiodinium* ITS2 OTU richness in seven coral genera collected from the Red Sea (RS), the Sea of Oman (SO), and the Persian/Arabian Gulf (PAG). Kruskal–Wallis post‐hoc *P*‐values of OTU richness given for pairwise comparisons between regions (significant *P*‐values < 0.05 in **bold**), average similarity (%) of the *Symbiodinium* OTU community within each coral host genus and region, SE = standard error, *ns* = not significant

Genus	Region	*n*	Mean no. of OTUs/sample (SE)	Max no. of OTUs/sample	Post‐hoc comparison OTU richness			no. of OTUs/genus	SIMPER (OTU community)
					RS vs. SO	RS vs. PAG	SO vs. PAG		Average similarity (%)
*Acropora*	RS	47	2.65 (0.10)	4	*ns*	*ns*	*ns*	9	87.02
*Acropora*	SO	22	2.45 (0.19)	4				7	56.08
*Acropora*	PAG	17	2.11 (0.24)	4				1	46.96
*Dipsastraea*	RS	11	2.36 (0.33)	5	*ns*	*ns*	*ns*	7	89.14
*Dipsastraea*	SO	15	2.60 (0.16)	3				7	57.24
*Dipsastraea*	PAG	17	2.29 (0.31)	5				9	78.76
*Montipora*	RS	16	2.68 (0.23)	5	*ns*	*ns*	*ns*	10	49.62
*Montipora*	SO	23	2.73 (0.24)	5				7	49.48
*Montipora*	PAG	8	2.00 (0.32)	3				5	46.96
*Pavona*	RS	10	2.90 (0.40)	6	*ns*	**0.025**	**0.010**	8	75.27
*Pavona*	SO	12	2.83 (0.24)	4				9	50.50
*Pavona*	PAG	16	1.68 (0.21)	4				6	80.27
*Pocillopora*	RS	35	2.74 (0.11)	4	**0.004**	*ns*	*ns*	8	84.63
*Pocillopora*	SO	17	1.88 (0.24)	4				7	88.49
*Pocillopora*	PAG	5	2.00 (0.31)	3				3	85.27
*Porites*	RS	24	5.62 (0.39)	9	*ns*	**< 0.001**	**< 0.001**	28	57.55
*Porites*	SO	35	5.00 (0.33)	10				19	62.51
*Porites*	PAG	21	2.14 (0.19)	4				10	82.79
*Stylophora*	RS	23	3.08 (0.23)	6	*ns*	*ns*	*ns*	10	69.44
*Stylophora*	SO	16	2.37 (0.20)	4				4	77.92
*Stylophora*	PAG	3	2.33 (0.33)	3				3	81.58
ALL		393	2.90 (0.07)	10	*ns*	**< 0.001**	**< 0.001**	92	87.02

**Figure 6:**
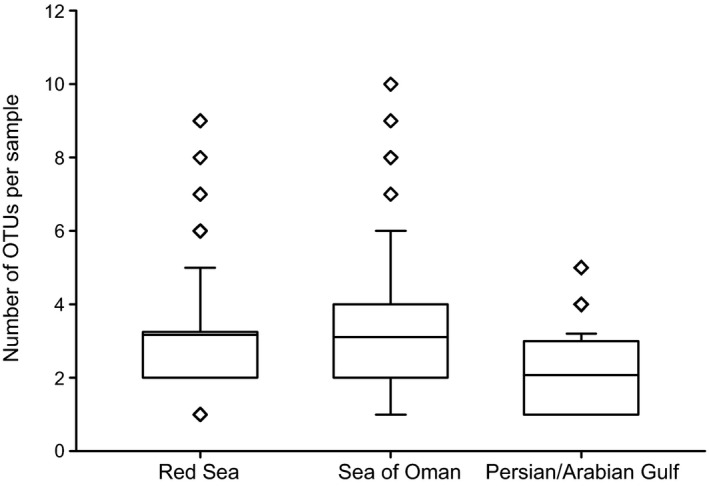
Box plot displaying the average number of OTUs per specimen collected from seven coral genera (*Acropora*,* Dipsastraea*,* Montipora*,* Pavona*,* Pocillopora*,* Porites*,* Stylophora*) in the Red Sea (*n* = 166), the Sea of Oman (*n *= 140), and the Persian/Arabian Gulf (*n* = 87). Center lines show the means; box limits indicate the 25th and 75th percentiles; whiskers extend to 5th and 95th percentiles, outliers are represented by diamonds.

OTU richness varied between coral host species and regions and ranged from an average of 1.68 OTUs per sample (*Pavona* in the PAG) to 5.62 OTUs (*Porites* in the RS) (Table 2). *Porites* colonies contained up to 10 OTUs and thus on average harbored roughly twice as many OTUs as all other genera. Furthermore, OTU richness of the seven host genera was significantly different between the three regions (Kruskal–Wallis, *H* = 40.29, *P *<* *0.001). On average, each specimen from the RS harboured 3.13 OTUs, compared to 2.86 OTUs in the SO and 2.18 OTUs in the PAG, respectively (Fig. 6). Furthermore, our analysis revealed a highly significant lower OTU richness in the PAG compared to the RS and the SO (both pairwise comparisons *P *<* *0.001), whereas OTU richness in the RS and SO were not significantly different from each other (Table 2). This overall pattern could be statistically reconstituted for individual host genera of *Pavona* and *Porites* and by trend for *Montipora* and *Acropora* (Table 2). For colonies of *Pocillopora* we found a lower OTU richness in the SO and in the PAG compared to the RS (but differences were only significant between the RS and SO, Table 2), and the same trend was apparent for *Stylophora* between the three regions. Differences in OTU richness between regions were not significant for *Dipsastraea* (Table 2).

## Discussion

This study represents a comprehensive survey on coral–*Symbiodinium* association using next‐generation sequencing techniques in one of the most extreme, but understudied geographical regions in the world. We investigated *Symbiodinium* diversity associated with almost 900 coral specimens encompassing 46 genera from the Red Sea, the Sea of Oman, and the Persian/Arabian Gulf. Given the large number of specimens collected, *Symbiodinium* diversity typing using next‐generation sequencing methodology was not only more convenient, but also allowed for the study of host–symbiont patterns including both sequence‐based ITS2 analysis and OTU‐based ITS2 diversity and richness analysis. A sequence‐based analysis permits direct assessment of coral‐associated symbionts (typically focusing on the more/most dominant members). Its reliance on previously recorded *Symbiodinium* types allows the comparison between studies and sites, but makes the assignment of provisionally new entities difficult. A benefit of the application of an OTU‐based framework may lie in its ability to estimate *Symbiodinium* diversity without the need for *a priori* and formal description of ITS2 symbiont types. Consequently, we used both approaches in this study in order to provide a comprehensive description of symbionts associated with corals of the Arabian Seas.

### Biogeography of *Symbiodinium* types around the Arabian Peninsula

Our analyses of host–*Symbiodinium* association in 46 coral genera across the Arabian Peninsula showed that coral–symbiont association was strongly defined by geographical location. Beyond the prevalence of regionally specific *Symbiodinium* types that distinguished the three regions, symbiont communities shifted from clade C and clade A dominance in the RS, over decreasing proportions of clade A and increasing proportions of clade D in the SO, to a *Symbiodinium* community dominated by clade D and to a lesser extent by clade C in the PAG. Not all coral genera could be sampled in all regions, yet sampling the most abundant taxa at each location enabled us to record the common *Symbiodinium* types in each region. In contrast to many other studies (LaJeunesse, 2002; LaJeunesse *et al*., 2004; Tonk *et al*., 2013), we found that coral host genus played a relatively small role in the identity of the dominant *Symbiodinium* type, but largely followed biogeographical patterns within most coral host genera around the Arabian Peninsula. Possible explanations for this observation could lie in the difference of environmental conditions between the three regions (see introduction), the comprehensive sampling approach over many diverging host genera, differences in sampling times between locations, or the use of highly resolving molecular techniques to characterize *Symbiodinium* associations.

A common feature of *Symbiodinium* communities in the Arabian Seas with those in the Indo‐Pacific and Atlantic‐Caribbean is the overproportionally high diversity in *Symbiodinium* clade C (LaJeunesse, 2005). This diversity can be attributed to a series of adaptive radiation events based on few ancestral *Symbiodinium* types, C1 and C3 (LaJeunesse, 2005), which were also present in our data set. Possible examples of regional diversification may be represented by *Symbiodinium* C41, which is separated from *Symbiodinium* C1 by a single base pair difference in the ITS2 sequence. *Symbiodinium* C41 was endemic, but regionally prevalent in the RS; and in the SO *Symbiodinium* C39 may represent another example of regional diversification. Moreover, fine‐scale genetic divergence within these types may not be fully resolved by the ITS2 marker, masking other potentially divergent lineages (Thornhill *et al*., 2014), as exemplified by the cryptic, but regionally prevalent C3–type species *Symbiodinium thermophilum* in the PAG (Hume *et al*., 2015, 2016), which likely also dominated the clade C assemblage observed in the southern PAG in this study (Hume *et al*., 2016).

### Insight into thermotolerant *Symbiodinium* types

Studying *Symbiodinium* diversity in one of the hottest regions where corals persist offers the opportunity to search for environmentally tolerant *Symbiodinium* types. Probably the most obvious such case lies in *S. thermophilum* in the PAG (Hume *et al*., 2015, 2016). *Porites* harbouring this *Symbiodinium* species were more resilient to heat stress than their Pacific counterparts harboring C15 (Hume *et al*., 2013). However, further investigation into this symbiosis has revealed a concordant adaptation to the high salinity of the PAG, deeming it unlikely that its thermal tolerance can be extended beyond its current range (D'Angelo *et al*., 2015). Furthermore, in the RS the main *Symbiodinium* type in *Porites* changed from C15 at cooler offshore locations to *Symbiodinium* D1a (and not C3) at warmer nearshore locations (Ziegler *et al*., 2015). Within the PAG, the northern reefs were distinct from the southern locations, mainly due to the higher prevalence of *Symbiodinium* from clade D in the north, an intriguing pattern that has been described before (Baker *et al*., 2004). While *Symbiodinium* from clade D may offer higher thermal tolerance, it may actually perform inferior compared to other types in supporting essential functions such as coral growth under non‐stressful conditions (Pettay *et al*., 2015). Beyond known heat‐resistant symbionts, we identified other *Symbiodinium* types prevalent around the Arabian Peninsula, whose tolerance to high temperature is presently unknown. For example, *Symbiodinium* type C41 and C39 were prevalent and restricted to the RS and the SO providing potential candidate endosymbiont types that display increased thermotolerance. Another source of heat‐resilient candidate endosymbionts may be derived from the identification of *Symbiodinium* types associated with coral genera that were stable between regions. For instance, *Stylophora* was commonly and dominantly associated with *Symbiodinium* A1 throughout the Arabian Peninsula, an association that is congruent with previous reports from the northern RS (LaJeunesse, 2001). By comparison, in other geographical locations *Stylophora* spp. are more commonly associated with *Symbiodinium* from clade C, such as in the Western Indian Ocean (LaJeunesse *et al*., 2010) and in the Great Barrier Reef (Sampayo *et al*., 2007; Stat *et al*., 2008b). This symbiont association is quite surprising, because outside the Caribbean, *Symbiodinium* from clade A are more commonly associated with non‐scleractinian taxa, such as zooanthids (Reimer *et al*., 2006), giant clams (Baillie *et al*., 2000), and jellyfish (LaJeunesse, 2001).

### *Symbiodinium* community composition and richness align with geographical regions and environmental settings

In addition to the biogeographical pattern of the most abundant *Symbiodinium* types between the three regions around the Arabian Peninsula, the composition of the *Symbiodinium* OTU community was more similar in the SO and the PAG compared to the RS. Commonly, *Symbiodinium* communities are structured along environmental gradients, for example, from nearshore to offshore locations across the shelf (Tonk *et al*., 2013) or over latitudinal temperature gradients (Loh *et al*., 2001; Macdonald *et al*., 2008). In light of the diverging environmental conditions between the SO and PAG, our observations suggest that the geographical proximity and thus the higher connectivity of populations between the two regions may be responsible for the higher similarity compared to the more distantly located RS.

Besides the similarities and differences in OTU community composition across geographical regions, we found distinct OTU richness patterns within corals depending on site and genus. For instance, coral colonies belonging to the genus *Porites* on average harbored twice as many *Symbiodinium* OTUs than corals from any other host genus; they also contained the largest number of OTUs encountered in a single colony by far. Coral colonies of *Porites* were found in association with a large diversity of other *Symbiodinium* types in addition to the two main types (C3 and C15), and the genus *Porites* roughly contained half of all recorded *Symbiodinium* OTUs in each of the three regions, but this may potentially be confounded by sampling of different (cryptic) species in this genus. In this regard, our observations contradict the notion of *Porites* as a symbiont specialist genus (Silverstein *et al*., 2012) and support the latest observations suggesting a large symbiont flexibility in *Porites* (Ziegler *et al*., 2015). Overall, corals from the PAG hosted the least diverse *Symbiodinium* communities compared to the RS and the SO, reflecting patterns observed in species diversity of coral hosts, fishes, and other reef‐associated fauna (Sheppard *et al*., 1992; Burt *et al*., 2011; Bauman *et al*., 2013). This might be due to the comparably young age of the PAG or the extreme environmental conditions with respect to high temperature variation as well as elevated salinity in the PAG constituting a selective bottleneck in which only highly specific host–symbiont pairings prevail (D'Angelo *et al*., 2015; Hume *et al*., 2015). Local environmental pressure may thus also be a limiting factor for diversity and distribution of both corals and *Symbiodinium* in this region (D'Angelo *et al*., 2015). At the same time, the biological significance of differences in symbiont richness over geographical regions and host genera is entirely unclear. For instance, it would be desirable to understand whether the increased OTU richness in *Porites* colonies constitutes an untapped resource of symbiont plasticity or provides a possible explanation for the environmental flexibility of this coral genus.

## Conclusions

This study utilized next‐generation sequencing of the ITS2 marker gene to analyze *Symbiodinium* composition associated with 46 coral genera and 892 specimens around the Arabian Peninsula. As such our study provides a comprehensive catalog and comparative assessment of symbiont diversity in this comparatively understudied region, where coral reefs thrive in one of the most extreme environmental settings on the planet. Our data show that corals around the Arabian Peninsula are associated with a large diversity of *Symbiodinium* types that are strongly structured by geographical location and to a lesser extent by coral host identity. The application of high‐resolution symbiont typing enabled the analyses of symbiont diversity in an OTU‐based framework that highlights differences in OTU richness associated with geographical region and host genus, the significance of which remain to be determined. In addition, our analysis highlights a set of potential thermotolerant *Symbiodinium* types outside clade D that warrant further investigation and emphasize that thermal tolerance is a species‐ or type‐specific, rather than clade‐specific trait.

## Data accessibility

Sequences determined in this study have been deposited in the NCBI Sequence Read Archive (http://www.ncbi.nlm.nih.gov/sra) under accession number PRJNA306572.

## Biosketch

The reef genomics lab at KAUST (https://reefgenomics.kaust.edu.sa) combines ecological, environmental, microbial, and molecular approaches to understand coral animal and reef ecosystem structure and function. The lab aims to develop an integrated understanding of the ecology and evolution of coral holobionts on a molecular and ecosystem level to predict adaptive capabilities and biotic response to environmental change.

## Supporting information


**Appendix S1** Sample overview.

**Appendix S2** 118,205 ITS2 sequences.

**Appendix S3** Proportion of sequences across samples.

**Appendix S4** Count table of sequences ≥ 5%.

**Appendix S5** Count table of OTUs.

**Appendix S6** Post‐hoc comparisons between reefs.
