# Supplementary material for: Biogeography and molecular diversity of coral symbionts in the genus Symbiodinium around the Arabian Peninsula
Source: J Biogeogr. 2017 Jan 2;44(3):674–86. doi: 10.1111/jbi.12913 (PMC5324606; doi:10.1111/jbi.12913)
Supplement: Supplementary file 7 [file JBI-44-674-s007.docx]

*Journal of Biogeography*

**SUPPORTING INFORMATION**

**Biogeography and molecular diversity of coral symbionts in the genus *Symbiodinium* around the Arabian Peninsula**

Maren Ziegler, Chatchanit Arif, John A. Burt, Sergey Dobretsov, Cornelia Roder, Todd C. LaJeunesse, Christian R. Voolstra

**Appendix S1** Overview over collection sites and samples, dates, sample ID, and sampling depth for 892 specimens.

**Appendix S2** Sequence file of 118,205 distinct *Symbiodinium* ITS2 sequences in 892 coral specimens collected around the Arabian Peninsula.

**Appendix S3** Proportions of 118,205 distinct *Symbiodinium* ITS2 sequences in 892 coral specimens collected around the Arabian Peninsula.

**Appendix S4** Abundance table of 223 *Symbiodinium* ITS2 sequences present with at least 5 % in at least one of the 892 coral samples collected around the Arabian Peninsula.

**Appendix S5** Abundance table of 92 *Symbiodinium* ITS2 OTUs recovered from 693 coral samples after subsampling to 1,000 reads per sample.

**Appendix S6** Post-hoc pairwise comparison of *Symbiodinium* OTU community structure between reefs sampled in each of three regions around the Arabian Peninsula (PERMANOVA).
